# Characterization of the colostrum proteome of primiparous Holstein cows and its association with colostrum immunoglobulin G concentrations

**DOI:** 10.1186/s40104-024-01144-y

**Published:** 2025-01-21

**Authors:** Ezequias Castillo-Lopez, Patrick Biber, Arife Sener-Aydemir, Karin Hummel, Ebrahim Razzazi-Fazeli, Nicole Reisinger, Qendrim Zebeli, Susanne Kreuzer-Redmer, Thomas Hartinger

**Affiliations:** 1 Center for Animal Nutrition and Welfare, University of Veterinary Medicine Vienna, Veterinärplatz 1, 1210 Vienna, Austria; 2 Christian Doppler Laboratory for Innovative Gut Health Concepts of Livestock, Vienna, Austria; 3 University of Veterinary Medicine Vienna, VetCore Facility (Mass Spectrometry), Vienna, Austria; 4 Dsm-Firmenich, Animal Nutrition & Health R&D Center, Tulln, Austria

**Keywords:** Bovine colostrum, Cow, Immunoglobulins, Passive immunity, Proteomics

## Abstract

**Background:**

The objective was to characterize the colostrum proteome of primiparous Holstein cows in association with immunoglobulin G (IgG) content. Immediately after calving, colostrum samples were collected from 18 cows to measure IgG concentration. Based on colostrum IgG content, samples were classified through cluster analysis and were identified as poor, average, and excellent quality. The proteome was assessed with quantitative shotgun proteomics; abundance data were compared among the colostrum types; enrichment analysis of metabolic processes and proteins classes was performed as well. We also tested correlations between this proteome and blood globulin level of cows and passive immunity level of calves.

**Results:**

On average, 428 proteins were identified per sample, which belonged mainly to cellular process, biological regulation, response to stimulus, metabolic process, and immune system process. Most abundant proteins were complement C3 (Q2UVX4), alpha-S1-casein (P02662), Ig-like domain-containing protein (A0A3Q1M032), albumin (A0A140T897), polymeric immunoglobulin receptor (P81265), lactotransferrrin (P24627), and IGHG1*01 (X16701_4). Colostrum of excellent quality had greater (*P* < 0.05) abundance of serpin A3-7 (A2I7N3), complement factor I (A0A3Q1MIF4), lipocalin/cytosolic fatty-acid binding domain-containing protein (A0A3Q1MRQ2), complement C3 (E1B805), complement component 4 binding protein alpha (A0AAF6ZHP5), and complement component C6 (F1MM86). However, colostrum of excellent quality had lower (*P* < 0.05) abundance of HGF activator (E1BCW0), alpha-S1-casein (P02662), and xanthine dehydrogenase/oxidase (P80457). This resulted in enrichment of the biological processes predominantly for complement activation alternative pathway, complement activation, complement activation classical pathway, humoral immune response, leukocyte mediated immunity, and negative regulation of endopeptidase activity in excellent-quality colostrum. Additionally, some colostrum proteins were found to be correlated with the blood globulin level of cows and with the passive immunity level of calves (*P* < 0.05; *r* ≥ 0.57).

**Conclusions:**

This study provides new insights into the bovine colostrum proteome, demonstrating associations between IgG levels and the abundance of other proteins, as well as the enrichment of metabolic processes related to innate immune response. Thus, results suggest that the colostrum proteomic profile is associated with the content of IgG. Future research should deeply explore the association of these findings with pre-calving nutrition status and blood composition of the cow, and with passive immunity transfer to the calf.

**Supplementary Information:**

The online version contains supplementary material available at 10.1186/s40104-024-01144-y.

## Introduction

Calves are born without acquired passive immunity because there is no transfer of immunoglobulin G across the placenta from the dam to the fetus [1]. Therefore, the consumption of colostrum during the first few hours of life, when immunoglobulin absorption efficiency is highest, is very important to guarantee passive immunity acquisition in the neonate [2]. Nonetheless, it has been reported that the amount of colostrum consumed or its quality (typically based on IgG content) is not always reflected in successful passive immunity acquisition in offsprings. Low passive immunity in calves may also contribute to increase mortality due to susceptibility to diseases [1]. Thus, besides IgG, there is a need for deeper assessment of other colostrum proteins or factors, which may affect the health of calves. Indeed, researchers [3] have summarized a variety of proteins that create a complex network programming the neonatal immune system in calves, including growth factors, cytokines, and chemokines.

On-farm evaluation of colostrum quality has typically relied on colostrometers, which are practical given their fast application. However, a colostrometer is a hydrometer that relates colostrum density to IgG concentration and cannot measure the concentration of specific proteins [4]. Additionally, the measurements obtained with colostrometers may be biased by the temperature of colostrum samples [5, 6]. Therefore, colostrum quality has also been determined with more precise laboratory methods, such as radial immunodiffusion [7], which is an accurate measurement for colostrum IgG [2]. This analysis, however, cannot detect other specific proteins that also influence colostrum quality or function. Because colostrum contains a plethora of less known proteins with most likely important roles in different metabolic pathways associated with the activation of the immune response [3, 8, 9], assessment of such proteins is crucial.

Therefore, the aim was to characterize the colostrum proteomic profile of primiparous Holstein cows and to evaluate the association between the content of colostrum IgG and its proteome. We also evaluated correlations between the colostrum proteome and the pre-calving blood globulin content of the cow as well as with the level of acquired passive immunity of the calf. We hypothesized that the content of colostrum IgG is strongly associated with the abundance of other immune-related proteins, thus, would be a suitable indicator for categorizing the proteome. We also expected to find correlations between the abundance of colostrum proteins and the content of blood globulins of the cow as well as with the acquired passive immunity of the calves.

## Materials and methods

This study is a side project from a larger study examining ruminal pH dynamics in dairy cows during the transition period and early lactation [10]. The experimental methods and protocols followed in this study were approved by the institutional animal care and use committee of University of Veterinary Medicine Vienna, and this was in accordance with the national authority of Austria according to the Animal Experiments Act, Tierversuchsgesetz (GZ: 2021-0.009.975).

### Animals, housing and maternity care

Eighteen primiparous Holstein cows were used (701 ± 89 kg initial body weight, and 3.4 ± 0.14 body condition score). All cows were raised together from younger age in the same farm. Due to differences in conception date, cows were enrolled in the study in a stepwise manner according to the expected calving date. During this study, cows were housed in a free-stall barn to allow normal herding behavior. The stall was equipped with individual deep litter cubicles with dimensions of 2.6 m × 1.25 m, and they were bedded with straw. Additionally, there was an area that was used for calving of approximately 68 m^2^ with straw bedding, where cows could rest or move freely. This area was located next to the individual cubicles and was close to the automatic feeders. Clean fresh water was available for ad libitum consumption.

Cows were closely monitored prepartum to detect signs of calving. If signs of difficulty were observed during labor, assistance was provided accordingly. Immediately after parturition, standard operating procedures postpartum were followed, which included physical examination, rehydration with energy drink supplement containing electrolytes, dextrose, and calcium (Rindavital Energietrunk, H. Wilhelm Schaumann GmbH & Co KG, Brunn am Gebirge, Austria), as well as first milking of the cows. Within 60 min after birth, colostrum was offered ad libitum to the calves. Calves were individually housed and fed twice daily with transition milk and mature milk from the corresponding dam; the feeding was conducted as commonly implemented at the farm by targeting 10% of calf body weight daily. Calves were weighed at birth and at 7 days of age.

### Diet, feeding management and feed analysis

Starting 3 weeks before expected parturition, cows were offered the same close-up diet following the nutritional recommendations of the Society of Nutrition Physiology [11]. This diet contained 38% grass silage, 30% corn silage, 32% concentrate, and 6.27 megajoules of net energy of lactation per kg dry matter (Table 1). Before the initiation of the experiment, cows were randomly assigned and trained to automatic feeders, so that each cow was allowed access to one independent feeder throughout the experiment using an ear tag transponder (Insentec B. V., Marknesse, Netherlands). Thus, the diets were independently given to each cow, and measurements of feed intake and related data were collected individually. The diets were prepared daily in the morning using a feed mixer (Trioliet Triomatic T15, Oldenzaal, Netherlands), and were offered as total mixed diets in the automatic feeders at 7:00. To ensure cows had ad libitum access to feed, feeders were refilled at 16:00 to target 10% refusals each day. Before the morning feeding, refusals were collected, weighed, and feed bins were cleaned every day. Samples of each feed ingredient as well as samples of the total mixed diets were collected 3 weeks before expected calving date and within the first week of lactation. Chemical analyses of feeds were performed as described previously [10]. Residual organic matter (OM) was calculated by partitioning non-fiber carbohydrates into starch and residual OM [12]. Particle size distribution of the diet fed was measured using the Penn state particle separator (PSPS) equipped with 3 screens (19.0, 8.0, and 1.18 mm) and a pan [13].

**Table 1:** Ingredients, chemical composition, and particle size fraction of the pre-partum diet fed to cows

Item	Content
Ingredients, % DM basis	
Grass silage	38.0
Corn silage	30.0
Close-up diet concentrate^a^	32.0
TMR chemical composition	
DM, % as fresh	46.6
Crude protein, %	15.2
Neutral detergent fiber, %	41.1
Acid detergent fiber, %	26.1
Starch, %	18.1
Non-fiber carbohydrates, %	35.7
Residual OM, %	17.6
Ether extract, %	2.29
Ash, %	7.10
NE_L_, MJ/kg DM	6.27
Particle fraction, % retained^b^	
Long	19.2
Medium	42.5
Short	35.3
Fine	2.98

^a^Close-up concentrate: The concentrate mixture contained (DM basis): barley meal (62.4%), rapeseed meal (20.0%), corn meal (9.0%), soybean meal (4.0%), vitamin and mineral supplement (3.5%), molasses (1.0%), magnesium oxide (0.1%)

^b^Particle fractions determined with the Penn State Particle Separator with a 19-mm screen (long), 8-mm screen (medium), 1.18-mm screen (short), and a pan (fine) [13]

### Determination of pre-calving blood proteins of cows

Blood samples were collected from the jugular vein of each cow three weeks before expected parturition using serum collection vacutainers (9 mL, Vacuette, Greiner Bio-One, Kremsmuenster, Austria). This sampling was performed before the first morning meal. To isolate the serum, blood samples were first stored at room temperature for approximately 1.5 h to allow clotting. Then, samples were centrifuged at 2,000 × *g* at 4 °C for 15 min (Centrifuge 5804 R, Eppendorf), the serum was pipetted into 2-mL tubes (Eppendorf) and stored at −80 °C.

Analysis was conducted after completion of the experiment. Briefly, blood concentrations of total proteins (kit total protein Gen.2, 03183734190, Roche Diagnostics, Basel, Switzerland) and albumin (kit albumin Gen.2, 03183688122, Roche Diagnostics, Basel, Switzerland) were evaluated at the Central Clinical Pathology Unit, University of Veterinary Medicine (Vienna, Austria). These analyses were performed with an automated autoanalyzer (Cobas 6000/c501; Roche Diagnostics) and the intra-assay variation was controlled by limiting the coefficient of variation to < 5% for these blood variables. Plasma globulin concentration was calculated as the difference between plasma total proteins and albumin concentrations [14].

### Determination of IgG in colostrum

Within 60 min after birth, colostrum samples were collected using sterile gloves for each sampling. Samples were collected from each pre-cleaned udder quarter after discarding the first 10 mL of colostrum. The colostrum was then pooled per cow and placed in sterile cryotubes that were immediately frozen at −80 °C for later analysis. At the end of the experiment, the IgG content was measured in samples.

The concentration of IgG was measured according to the method previously described using Single Radial Immunodiffusion [7] with the GTP Immuno Bov IgG IDRing Test (Issoire, France). Briefly, a standard curve with concentrations of 25, 50, 100 and 200 µg/mL of IgG was prepared. The colostrum samples were thawed and diluted to a ratio of 1:750. This dilution was conducted in 3 steps: dilution #1 was prepared in a ratio of 1:10 (colostrum to physiologic water containing 0.51 mol/L NaCl, respectively), then dilution #2 was prepared in ratio of 1:10 (dilution #1 to SRID buffer, respectively), and dilution #3 was prepared in a ratio of 1:7.5 (dilution #2 to SRID buffer, respectively). Then, 15 µL of each diluted sample were dispensed in the wells. Unused wells were filled with deionized water. Diffusion was then conducted between 16 and 20 h in an incubator at 35 ± 5 °C. The diffusion step was stopped by adding 5 mL of a 2% acetic acid solution, and samples were incubated at room temperature for 1 min. Then, the plate was rinsed twice with deionized water; to do so, 5 mL of water were used, and the plate was incubated between 10 and 15 min at room temperature. The diameters of precipitates were measured visually on a SRID Plate reader (IDRing Viewer-S, GTP Immuno, Issoire, France). The standard curve is established by plotting the square root concentration on the abscissa against the diameters of the precipitates for each of standard on the ordinate. Then, the concentration of IgG for each sample was calculated with Microsoft Excel using the standard curve as a reference.

### Classification of colostrum samples based on IgG content

**Fig. 1:**
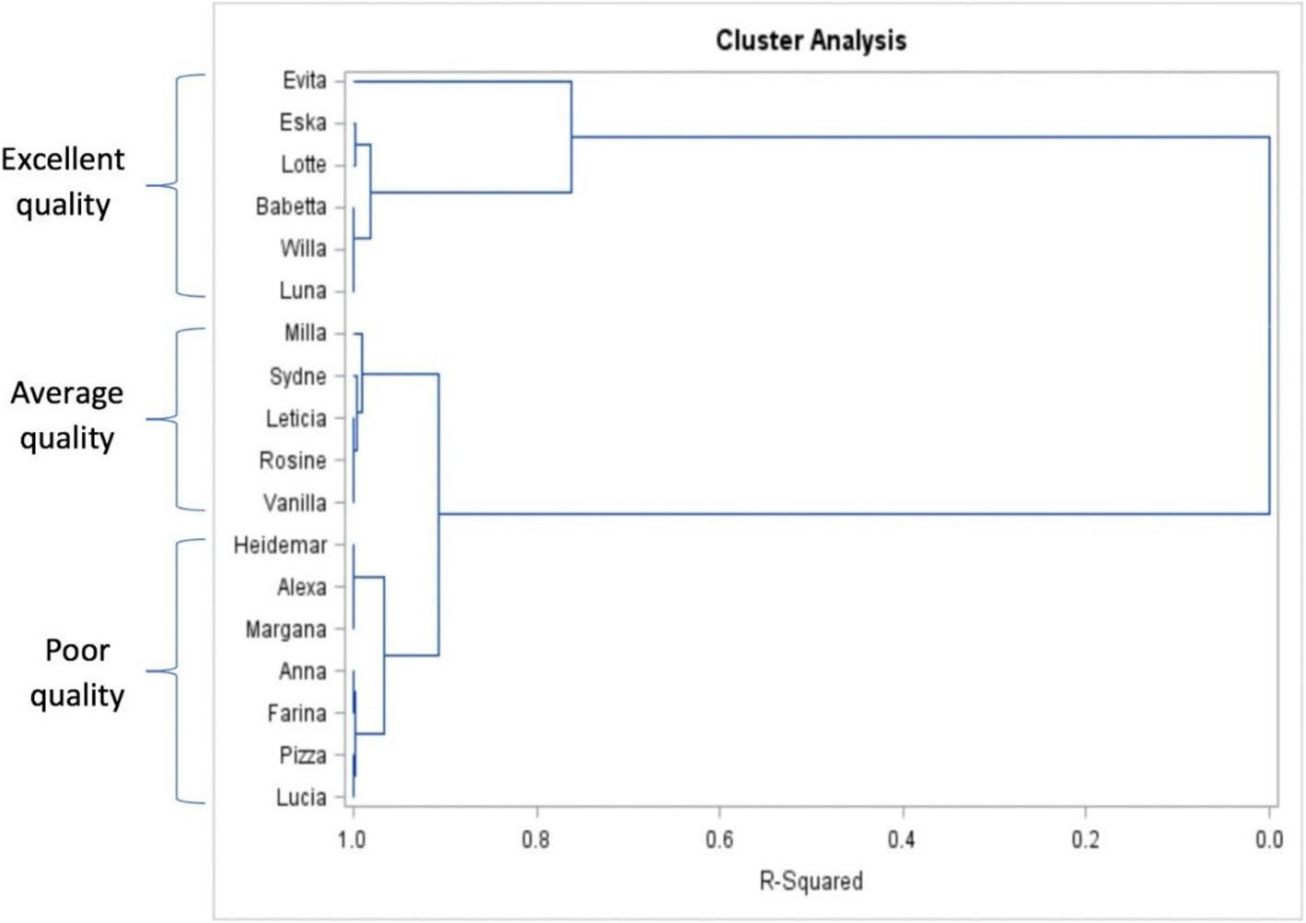
Hierarchical cluster dendrogram showing the 3 clusters according to colostrum quality, constructed using the concentration of IgG as an input. Samples with lower content of IgG (IgG ≤ 41.82 mg/mL, Poor quality, *n* = 7), medium content of IgG (41.82 < IgG ≤ 52.78 mg/mL, Average quality, *n* = 5) and high content of IgG (IgG ≥ 64.68 mg/mL, Excellent quality, *n* = 6). The *y*-axis shows the names of the cows used in the study

Colostrum samples were divided according to quality based on the content of IgG using a cluster analysis in SAS under the Euclidean distance metric using proc distance and Ward’s minimum variance method with proc cluster in SAS (SAS Institute Inc., Cary, NC, USA). The samples were plotted against the R-squared, which is a value that explains the proportion of variance accounted for by the clusters (Fig. 1). Then, we named the colostrum groups based on the results of the cluster analysis, which identified 3 groups: 1) cluster poor quality, *n* = 7; which included samples with lower content of IgG (IgG ≤ 41.82 mg/mL), 2) cluster average quality, *n* = 5; which included samples with medium content of IgG (41.82 < IgG ≤ 52.78 mg/mL), and 3) cluster excellent quality, *n* = 6; which included samples with high content of IgG (IgG ≥ 64.68 mg/mL).

### Estimation of acquired passive immunity in calves

Similarly to other reports [15, 16], at 1 week of age, blood samples were taken from the jugular vein of each calf using a vacutainer (9 mL, Vacuette, Greiner Bio-One, Kremsmuenster, Austria) and processed to obtain the blood serum by centrifugation at 2,000 ×* g* at 4 °C for 15 min. Then, calf acquired passive immunity was estimated through Brix refractometry (Kerbl Austria Handels GmbH, Poggersdorf, Austria) that estimates passive immunity in the calves based on the Brix %, which has been shown to be highly correlated with serum total protein in calves [17–20]. For each analysis, measurements were performed in duplicate, then the average of the technical replicate were calculated.

### Sample preparation for colostrum proteomic analysis

Initially, whole colostrum samples were thawed and prepared by aliquoting 1 mL into 1.5-mL microcentrifuge tubes, with one aliquot per sample proceeding to analysis and the rest stored at −80 °C. Each thawed selected aliquot received 10 µL of proteinase inhibitor cocktail (Sigma-Aldrich, Vienna, Austria) to prevent protein degradation. Subsequently, these aliquots were diluted in 9 mL of cold 1× PBS in 15-mL Falcon tubes (Sarstedt, Austria), ensuring thorough mixing. After dilution, the samples underwent centrifugation step at 3,350 × *g* for 30 min at 4 °C to separate the whey from cellular components and fat. Fat at the top phase was carefully removed using a sterile spatula and the remaining supernatant was collected and divided into 1.2 mL aliquots and stored at −80 °C for further analysis. One aliquot was used to assess the quality and integrity of the extracted proteins using SDS-PAGE.

The protein concentration was determined using a spectrophotometer with the Pierce 660 nm reagent according to the manufacturer’s protocol (DS-11 FX+ , DeNovix Inc., USA). For further processing, 30 μg of the protein sample were filled up to 500 μL with 8 mol/L urea in 50 mmol/L TRIS and were loaded on to a Pall 10 kDa ultrafiltration device (Fisher Scientific GmbH, Schwerte, Germany). The solution was centrifuged 2 times for 20 min at 10,000 × *g*. The proteins were reduced with 200 mmol/L DTT (37 °C, 30 min) and alkylated with 500 mmol/L iodoacetamide (IAA; 37 °C, 30 min) on the filter. After washing the samples twice with 100 μL 50 mmol/L TRIS, digestion was carried out using Trypsin/LysC Mix in a ratio of 1:25 protease:protein overnight. Digested peptides were recovered with 3 × 50 μL of 50 mmol/L TRIS and acidified with 1 μL concentrated trifluoroacetic acid (TFA). Before nanoLC-MS/MS analysis, peptide extracts were desalted and cleaned using C18 spin columns (Pierce) according to the manufacturer’s protocol. The dried peptides were redissolved in 300 μL 0.1% TFA, of which, 5 μL were injected to the nanoLC-MS/MS system.

### Proteomic analysis and liquid chromatography-mass spectrometry (nanoLC–MS/MS)

Proteomic analysis was conducted as previously detailed by our research group in Castillo-Lopez et al. [21]. Briefly, this was performed on a nanoLC-MS/MS system consisting of a nano-HPLC Ultimate 3000 RSLC (Thermo Scientific Dionex) directly coupled to a high-resolution Q Exactive HF Orbitrap mass spectrometer (Thermo Scientific) using a nano-ESI ion source. Peptides were preconcentrated on a 5-mm Acclaim PepMap μ-Precolumn (300 μm inner diameter, 5 μm particle size, and 100 Å pore size, Thermo Scientific Dionex) before being separated on a 25-cm Acclaim PepMap C18 column (75 μm inner diameter, 2 μm particle size, and 100 Å pore size, Thermo Scientific Dionex). The mobile phase for sample loading was 2% acetonitrile (ACN) in ultrapure H_2_O with 0.05% TFA with a flow rate of 5 μL/min, whereas for peptide separation gradient elution with a flow rate of 300 nL/min was performed. The gradient started with 4% B (80% ACN with 0.08% formic acid) for 7 min, increased to 31% in 60 min and to 44% in additional 5 min. A washing step with 95% B followed. Ultrapure H_2_O with 0.1% formic acid was used as a mobile Phase A.

The MS full scans were acquired between the ranges *m/z* 350–2,000 Da with a resolution of 60,000. The maximum injection time was 50 ms and the automatic gain control was set to 3e6. The top 10 most intense ions were further fragmented in the Orbitrap via higher-energy collision dissociation activation over a mass range between *m/z* 200 and 2,000 Da with a resolution of 15,000 and an intensity threshold of 4e3. Ions with a charge state +1, +7, +8 and > +8 were excluded. Normalized collision energy was set at 28. The automatic gain control was set at 5e4 and the maximum injection time was 50 ms. In order to avoid repeated peak fragmentation dynamic exclusion of precursor ion masses over a time window of 30 s was used. The database search was performed using the Proteome Discoverer Software 3.1.1.93 (Thermo Fisher Scientific). The protein database was downloaded from the reference *Bos taurus* proteome from the UniProt homepage, downloading one protein sequence per gene (https://www.uniprot.org/proteomes/UP000009136; download date 11.11.2024; 26,940 sequences). In addition to the UniProt database, the common contaminant database cRAP was used (https://www.thegpm.org/crap/). Furthermore, a custom database (CustomDB_Immunoglobulins_Bos taurus_241111.fasta) was included as well; to do so, the bovine heavy (IGHC), light lambda (IGLC) and light kappa (IGKC) constant region sequences were downloaded from https://www.imgt.org/vquest/refseqh.html, the sequences were checked so that each have a unique identification. Search settings were as follows: 10 ppm precursor mass tolerance and 0.02 Da fragment mass tolerance; dynamic modifications allowed were oxidation of methionine, deamination of N and Q, Gln to pyro-Glu, and static modification carbamidomethylation on cysteine. Only proteins with at least two identified peptides were reported. The label free quantification strategy was applied to compare protein abundance in the experiments. Additionally, the protein IDs that resulted as “uncharacterized” or identified as “Ig-like domain-containing protein” were further examined with the Basic Local Alignment Search Tool (BLAST) of the National Center for Biotechnology Information (NCBI) to verify whether they can be identified. Details as well as information for analysis of each technical replicate, protein identification and number of peptides as well as the compiled raw abundance data of proteins detected for all samples and cows can be found in the file deposited on Mendeley Data [22].

### Functional enrichment

The list of the protein IDs that composed the bovine colostrum core proteome was used to determine the Gene Ontology (GO) terms as well as the protein classes that are represented in the colostrum proteome using Protein Analysis Through Evolutionary Relationships (PANTHER) classification tool (http://www.pantherdb.org/) [23].

To gain a deeper understanding on the functions of the differentially abundant proteins, functional enrichment of the protein–protein interaction (PPI) networks were obtained using STRING [24].

### Bioinformatics and statistical analysis

After calculating the median between technical replicates, all missing values were replaced by 0 and imported into RStudio [25] for analysis. The level of concordance between technical replicates was evaluated by hierarchical cluster analysis using the “ward.D2” method (Additional file 1). As part of the quality control, filtering of data was performed to ensure that only robust measurements and quantifications of the core proteome are used for data analysis. To do so, filtering was performed based on the coefficient of variation of raw abundance data using the suggested default cutoff value of 25% [26]. Differential abundance of proteins was analyzed in RStudio as we have previously described [21]. Only proteins that were present in both technical replicates of each sample were considered for further analysis. Differences between groups were considered significant when the Benjamini-Hochberg (FDR) adjusted *P* values were < 0.05 [27], and a tendency when 0.05 ≥ *P* < 0.1.

Differential abundance analysis was conducted to evaluate the effect of the content of colostrum IgG on the proteome. Log2- transformed values were used to calculate the fold difference. Correlation analysis based on Pearson correlation coefficients was conducted with SAS (SAS Institute Inc., Cary, NC, USA) to evaluate the association between abundance of colostrum proteins and cow’s pre-calving blood globulin content. In addition, the correlation between colostrum proteins and estimates of passive immunity of calves was evaluated. Then, correlation networks were constructed in RStudio using packages igraph v1.2.7 and ggraph v2.0.5 to show associations with significant correlations (*r* ≥|0.57| and *P* < 0.01).

## Results

### Overall colostrum proteome and most abundant proteins

**Fig. 2:**
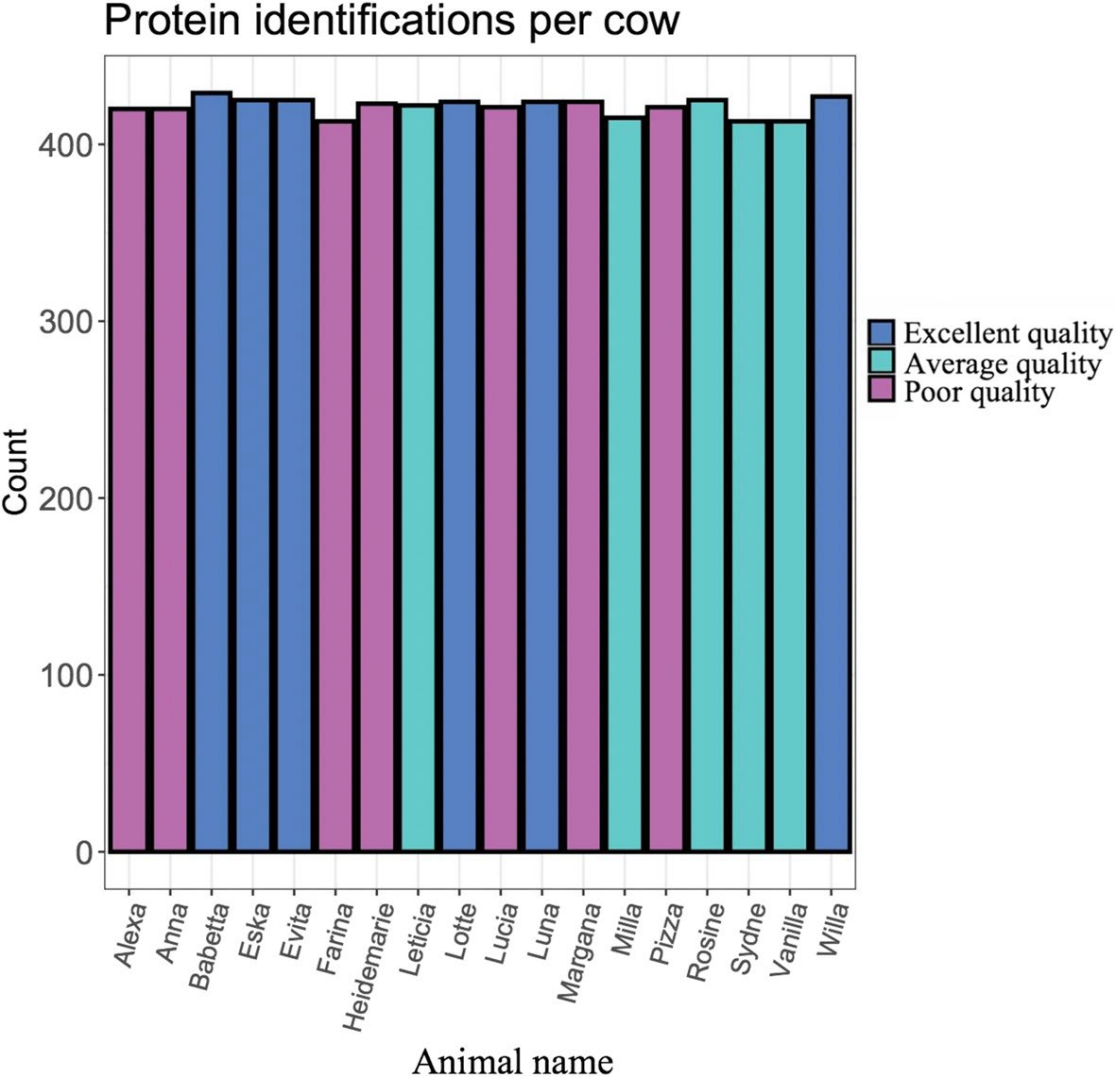
Number of proteins identified (> 2 peptides) in the colostrum samples of primiparous Holstein cows used in this experiment

A total of 461 proteins were identified across all samples, the average number of identified proteins with at least 2 peptides was 428 per sample, with a minimum of 419 and a maximum of 437 per sample (Fig. 2). The proteins identified with the highest numbers of identified peptides mapped to the colostrum proteome are listed in Table 2. The top 10 of these proteins included complement C3 OS = *Bos taurus* (Q2UVX4), alpha-S1-casein OS = *Bos taurus* (P02662), Ig-like domain-containing protein OS = *Bos taurus* (A0A3Q1M032), albumin OS = *Bos taurus* (A0A140T897), polymeric immunoglobulin receptor OS = *Bos taurus* (P81265), lactotransferrin OS = *Bos taurus* (P24627), IGHG1*01 *Bos taurus* (X16701_4), IGHG1*04 *Bos taurus*_Holstein (KT723008_45), milk fat globule EGF and factor V/VIII domain containing OS = *Bos taurus* (F1MXX6), and lipocalin/cytosolic fatty-acid binding domain-containing protein OS = *Bos taurus* (A0AAF6Z0B1). For all purposes, only those proteins with ≥ 2 peptides were taken into account.

**Table 2:** Most abundant proteins detected in the proteome of colostrum of the primiparous Holstein cows

ID	Protein name^a^
Q2UVX4	Complement C3 OS = *Bos taurus*
P02662	Alpha-S1-casein OS = *Bos taurus*
A0A3Q1M032	Ig-like domain-containing protein OS = *Bos taurus*
A0A140T897	Albumin OS = *Bos taurus*
P81265	Polymeric immunoglobulin receptor OS = *Bos taurus*
P24627	Lactotransferrin OS = *Bos taurus*
X16701_4	IGHG1*01 *Bos taurus*
KT723008_45	IGHG1*04 *Bos taurus*
F1MXX6	Milk fat globule EGF and factor V /VIII domain containing OS = *Bos taurus*
A0AAF6Z0B1	Lipocalin/cytosolic fatty-acid binding domain-containing protein OS = *Bos taurus*
A0A452DI34	Alpha-lactalbumin OS = *Bos taurus*
P02663	Alpha-S2-casein OS = *Bos taurus*
A0A452DHW7	Beta-casein OS = *Bos taurus*
KT723008_79	IGHM2*02 *Bos taurus*
F1MH40	Ig-like domain-containing protein OS = *Bos taurus*
A0A140T8A9	Kappa-casein OS = *Bos taurus*
P01888	Beta-2-microglobulin OS = *Bos taurus*

^a^Most abundant proteins was based on the number of identified peptides mapped to the protein databases

### Biological processes, protein classes and molecular functions detected in colostrum

**Fig. 3:**
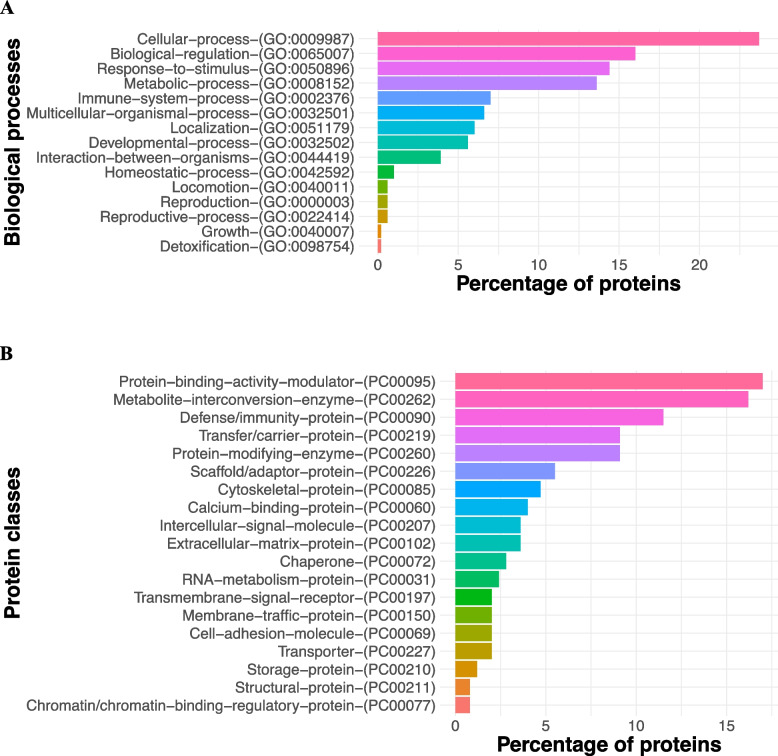
Major biological processes and protein calsses in the colostrum samples.** A** Biological processes enriched in the colostrum samples of primiparous Holstein cows used in this experiment. **B** Protein classes found in the colostrum samples

The analysis with PANTHER showed that the colostrum proteome included 15 biological processes (Fig. 3A). The top 10 of these processes were: cellular process (GO:0009987), biological regulation (GO:0065007), response to stimulus (GO:0050896), metabolic process (GO:0008152), immune system process (GO:0002376), multicellular organismal process (GO:0032501), localization (GO:0051179), developmental process (GO:0032502), biological process involved in interaction between organisms (GO:0044419), and homeostatic process (GO:0022414).

The analysis with PANTHER also revealed that the colostrum proteome included 19 protein classes (Fig. 3B). The most predominant of these categories were: protein-binding activity modulator (PC00095), metabolite interconversion enzyme (PC00262), defense/immunity protein (PC00090), transfer/carrier protein (PC00219), protein modifying enzyme (PC00260), scaffold/adaptor protein (PC00226), cytoskeletal protein (PC00085), calcium-binding protein (PC00060), intercellular signal molecule (PC00207) and extracellular matrix protein (PC00102) and chaperone (PC00072).

Additionally, 10 molecular functions were identified within the colostrum proteome, which were: binding (GO:0005488), catalytic activity (GO:0003824), molecular function regulator activity (GO:0098772), structural molecule activity (GO:0005198), transporter activity (GO:0005215), molecular transducer activity (GO:0060089), antioxidant activity (GO:0016209), molecular adaptor activity (GO:0060090), cargo receptor activity (GO:0038024), and transcription regulator activity (GO:0140110).

### Associations between colostrum IgG content on the proteomic profile

**Fig. 4:**
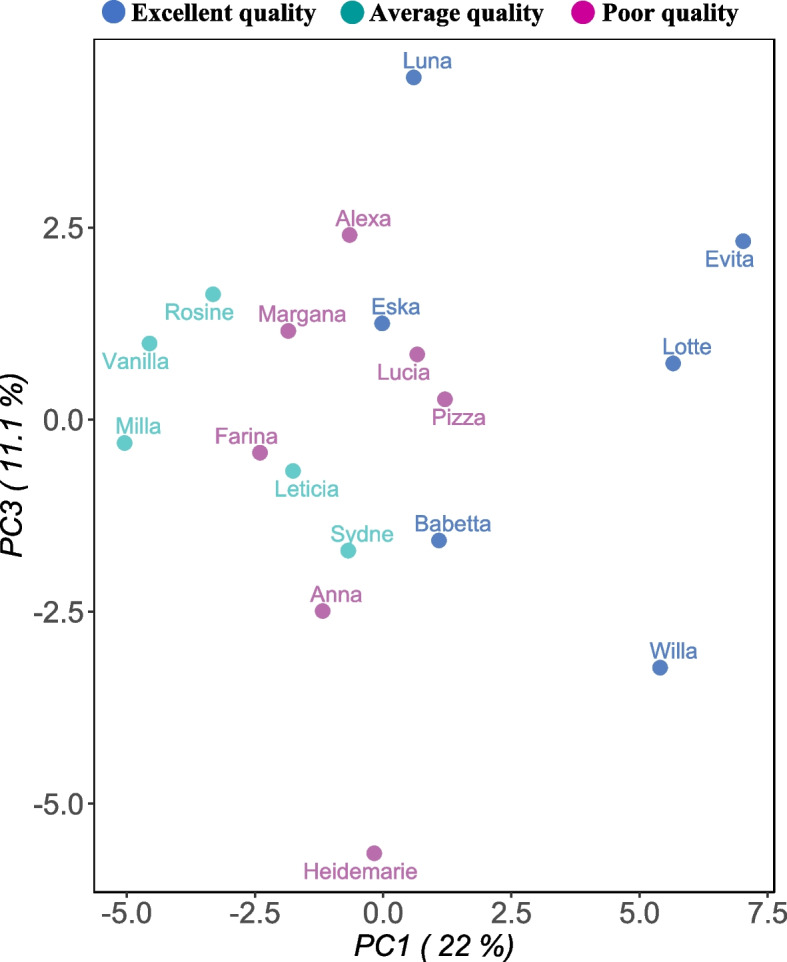
Principal component analysis plot showing clustering of the colostrum samples (represented by the name of cow) according to level of IgG content

Principal component analysis of the colostrum proteome allowed identification of possible sources of variations. Figure 4 shows grouping of colostrum samples, according to the level of IgG content, thus colostrum quality. Overall, this indicates that the different concentration of IgG has an influence on the proteomic profile among samples. Principal component 1 is represented on the *x*-axis, with 22% variation and principal component 3 on the *y*-axis with 11.1% variation.

More specifically, the differential abundance and fold change analysis revealed that 33 proteins differed or tended to be different according to colostrum quality (Table 3**)**. Most of these differences were found when comparing the average quality vs. excellent quality colostrum types. For example, some of those found in greater abundance in colostrum of excellent quality include primary amine oxidase, lung isozyme OS = *Bos taurus* (O46406), complement C3 OS = *Bos taurus *(E1B805), complement component 4 binding protein alpha OS = *Bos taurus* (A0AAF6ZHP5), serpin A3-7 OS = *Bos taurus* (A2I7N3), extracellular matrix protein 1 OS = *Bos taurus* (A0A3S5ZPB5), complement subcomponent C1r OS = *Bos taurus* (A5D9E9), complement component C6 OS = *Bos taurus* (F1MM86), kinesin-like protein OS = *Bos taurus* (A0AAA9SA93), CD5 molecule like OS = *Bos taurus* (A6QNW7), serpin domain-containing protein OS = *Bos taurus *(A0A3Q1MGZ6), lipocalin/cytosolic fatty-acid binding domain-containing protein OS = *Bos taurus* (A0A3Q1MRQ2), complement C8 alpha chain OS = *Bos taurus* (F1MX87), complement factor I OS = *Bos taurus* (A0A3Q1MIF4), and prothrombin OS = *Bos taurus* (A0A452DI66). On the other hand, some of the proteins found in lower abundance in colostrum of excellent quality included selenoprotein F OS = *Bos taurus* (A8YXY3), kappa-casein OS = *Bos taurus* (A0A140T8A9), xanthine dehydrogenase/oxidase OS =* Bos taurus* (P80457), beta-casein OS = *Bos taurus* (A0A452DHW7), alpha-S1-casein OS = *Bos taurus* (P02662), HGF activator OS = *Bos taurus* (E1BCW0).

**Table 3:** Differences in the colostrum proteome of samples with different quality (content of IgG)

Protein ID	Protein name	Colostrum quality^a^		SEM^b^	Log_2_diff^c^	*P*-value^*^
		Excellent quality	Average quality			
A2I7N3	Serpin A3-7 OS = *Bos taurus*	24.827	23.847	0.123	-0.98	0.029
A0A3Q1MIF4	Complement factor I OS = *Bos taurus*	26.81	26.154	0.103	-0.656	0.033
A0A3Q1MRQ2	Lipocalin/cytosolic fatty-acid binding domain-containing protein OS = *Bos taurus*	23.796	23.015	0.117	-0.781	0.033
E1B805	Complement C3 OS = *Bos taurus*	21.969	20.816	0.191	-1.153	0.033
E1BCW0	HGF activator OS = *Bos taurus*	18.708	20.846	0.311	2.138	0.033
A0AAF6ZHP5	Complement component 4 binding protein alpha OS = *Bos taurus*	24.792	23.797	0.182	-0.995	0.035
F1MM86	Complement component C6 OS = *Bos taurus*	26.314	25.426	0.147	-0.888	0.035
P02662	Alpha-S1-casein OS = *Bos taurus*	35.283	35.784	0.119	0.501	0.054
A6QNW7	CD5 molecule like OS = *Bos taurus*	30.315	29.467	0.169	-0.848	0.055
P80457	Xanthine dehydrogenase/oxidase OS = *Bos taurus*	28.361	28.72	0.113	0.359	0.055
O46406	Primary amine oxidase, lung isozyme OS = *Bos taurus*	18.081	16.706	0.297	-1.375	0.056
A0AAA9SA93	Kinesin-like protein OS = *Bos taurus*	17.82	16.966	0.187	-0.854	0.07
A0A452DHW7	Beta-casein OS = *Bos taurus*	34.514	34.875	0.104	0.361	0.072
A5D9E9	complement subcomponent C1r OS = *Bos taurus*	24.185	23.279	0.21	-0.906	0.072
A0A452DI66	Prothrombin OS = *Bos taurus*	28.18	27.75	0.087	-0.43	0.087
A8YXY3	Selenoprotein F OS = *Bos taurus*	24.161	24.364	0.079	0.203	0.087
F1MX87	Complement C8 alpha chain OS = *Bos taurus*	24.742	24.023	0.161	-0.719	0.087
A0A140T8A9	Kappa-casein OS = *Bos taurus*	33.913	34.259	0.14	0.346	0.089
A0A3Q1MGZ6	Serpin domain-containing protein OS = *Bos taurus*	26.607	25.79	0.207	-0.817	0.089
A0A3S5ZPB5	Extracellular matrix protein 1 OS = *Bos taurus*	21.683	20.706	0.264	-0.977	0.089
A0A3S5ZPM3	6-phosphogluconate dehydrogenase, decarboxylating OS = *Bos taurus*	19.122	19.574	0.145	0.452	0.089
A2I7N1	Serpin A3-5 OS = *Bos taurus*	26.348	25.774	0.135	-0.574	0.089
E1BA17	Collagen type XIV alpha 1 chain OS = *Bos taurus*	26.707	27.204	0.184	0.497	0.089
G3N0S9	Sushi domain-containing protein OS = *Bos taurus*	25.618	24.91	0.179	-0.708	0.089
P81187	Complement factor B OS = *Bos taurus*	28.164	27.738	0.09	-0.426	0.089
Q2KIX7	Protein HP-25 homolog 1 OS = *Bos taurus*	26.127	25.443	0.162	-0.684	0.089
Q2UVX4	Complement C3 OS = *Bos taurus*	31.666	31.103	0.126	-0.563	0.089
Q5EAB4	Chitinase domain-containing protein 1 OS = *Bos taurus*	22.888	23.137	0.112	0.249	0.089
Q29437	Primary amine oxidase, liver isozyme OS = *Bos taurus*	26.615	25.739	0.215	-0.876	0.092
A0AAA9SQF7	Joining chain of multimeric IgA and IgM OS = *Bos taurus*	28.355	27.576	0.206	-0.779	0.099
A0A3Q1MEM9	Mucin-1 OS = *Bos taurus*	22.143	22.763	0.244	0.62	0.101
A0A3Q1MJT2	Alpha-1-B glycoprotein OS =* Bos taurus*	28.346	27.834	0.129	-0.512	0.101
		Excellent quality	Poor quality			
P02662	Alpha-S1-casein OS = *Bos taurus*	35.283	35.667	0.081	0.384	0.056
P02663	Alpha-S2-casein OS = *Bos taurus*	33.523	34.112	0.121	0.589	0.056

^a^Poor quality: < 42 mg of immunoglobulins/mL of colostrum; Average quality: between 44 and 54 mg of immunoglobulins/mL of colostrum; Excellent quality: > 64 mg of immunoglobulins/mL of colostrum

^b^Standard error of the mean

^c^Fold 2 differences between the means being compared

^*^P-values are adjusted with the Benjamini-Hochberg method

When comparing colostrum samples of excellent and poor quality, the protein alpha-S1-casein OS = *Bos taurus* (P02662), and protein alpha-S2-casein OS = *Bos taurus* (P02663) were found in lower abundance in colostrum samples of excellent quality.

### Protein interaction and functional enrichment of differentially abundant proteins

**Fig. 5:**
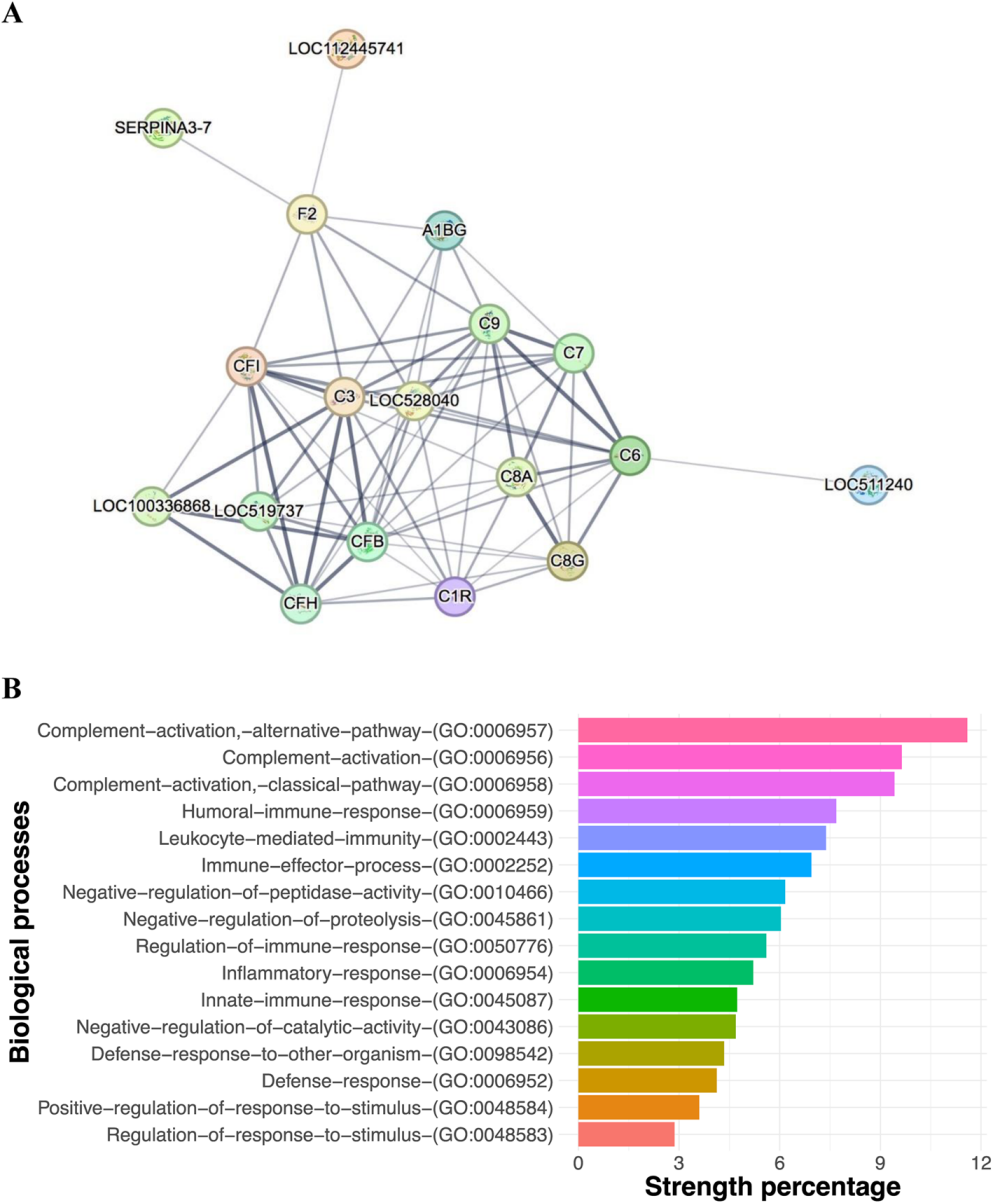
Major protein interactions and biological processes in colostrum of excellent quality.** A** Protein to protein interaction for proteins (identifications shown) that differed between colostrum samples. **B** Major protein classes that were significantly enriched (*P* < 0.05), which show the percentage of proteins for each protein class

To gain a deeper understanding on the functions of the differentially abundant proteins in the colostrum samples of excellent, functional enrichment of the protein–protein interaction (PPI) networks was obtained using STRING. The network interaction showed associations primarily amongst serpins and complement components, as well as component cofactors (Fig. 5A). Sixteen biological processes were found to be significantly enriched (*P* < 0.05) in the protein–protein network in the colostrum of excellent quality (Fig. 5B). The main biological processes enriched biological processes mainly for complement activation, alternative pathway (GO:0006957), complement activation (GO:0006956), complement activation classical pathway (GO:0006958), humoral immune response (GO:0006959), leukocyte mediated immunity (GO:0002443), and negative regulation of endopeptidase activity in excellent-quality colostrum (GO:0010466).

### Correlation of colostrum proteins with cow’s blood globulin and calf’s passive immunity

**Fig. 6:**
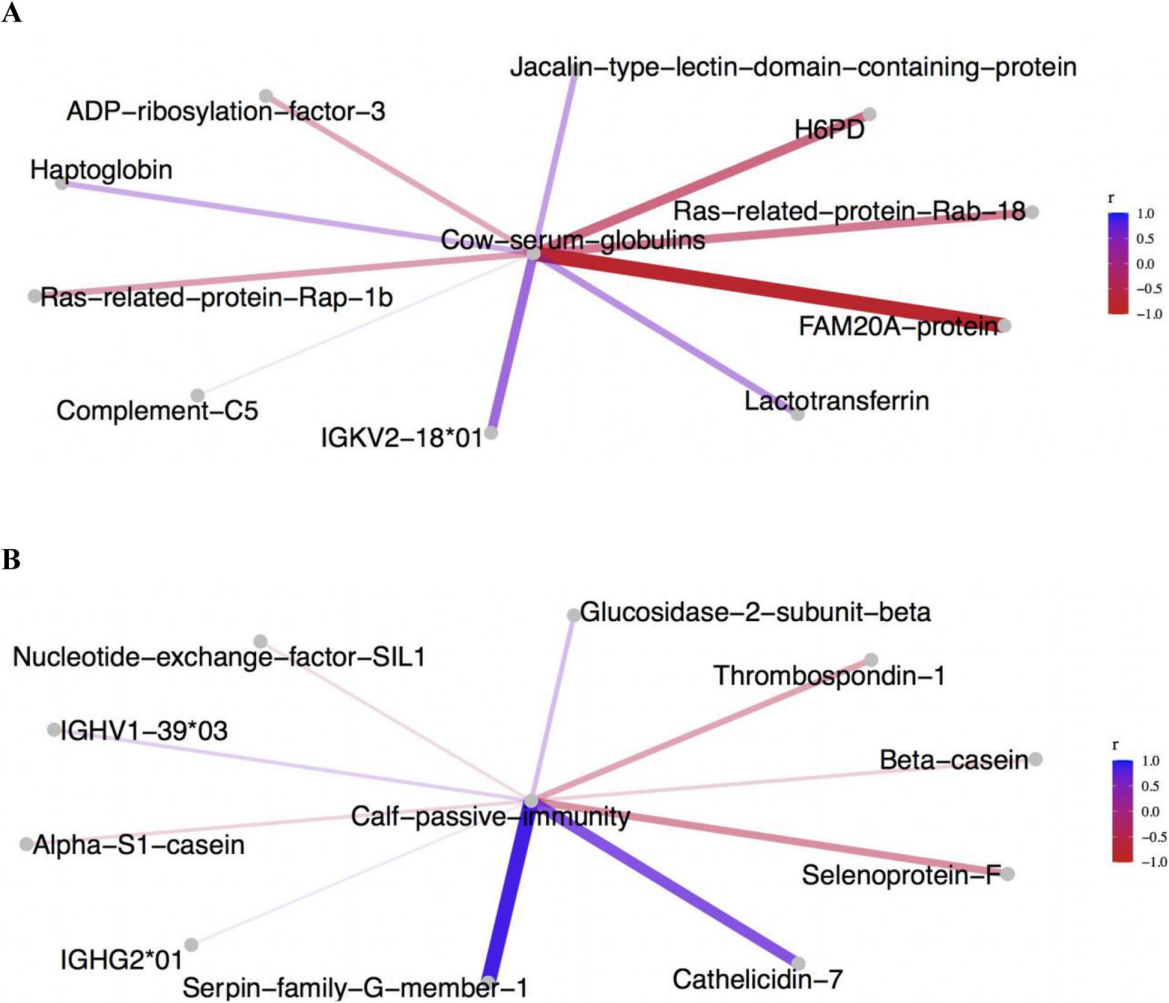
Correlations of colostrum proteins with cow serum globulins and with calf immunity.** A** Correlations between pre-calving cows’ blood globulin content and proteins in colostrum (*P* < 0.01). **B** Correlations between calf acquired passive immunity and abundance of proteins in consumed colostrum (*P* < 0.01)

The top 10 correlation between the content of globulins in the blood of cows pre-calving and colostrum proteins is shown in Fig. 6A. For example, positive correlations (*P* < 0.05;* r* =|0.60|) were found haptoglobin (Q2TBU0), lactotransferrin (P24627) and IGKV-18*01 (IMGT000047_11), but negatively correlated with ras-related protein rab-18 (Q0IIG8) and with hexose-6-phosphate dehydrogenase/glucose 1-dehydrogenase (A0A3Q1LRN7).

First colostrum intake averaged 1.97 ± 0.56 kg; calf birth weight at calving and in week 1 of age averaged 41.33 ± 4.19 and 49.87 ± 5.59 kg, respectively. The estimation of acquired passive immunity with refractometry indicated values of Brix % that ranged from 8.7 to 9.85, with an average of 9.2 ± 0.37. Significant positive correlations (*r* = 0.60) were found between calf’s passive immunity and the abundance of serpin family G member 1 (A0AAA9TZ37), cathelicidin (P56425), and with IGHG2*01 (M36946_1; *P* < 0.01; Fig. 6B).

## Discussion

Yet, the bovine colostrum proteome and factors affecting its profile have not been completely characterized. Thus, the goal of this study was to gain further insights into the colostrum proteome of primiparous Holstein cows. Previous research has revealed the presence of 86 [9], 95 [28] and 162 [29] proteins in bovine colostrum in both primiparous and multiparous Holstein cows. More recently, 471 proteins were found in the colostrum, and in milk samples collected during the first five days of lactation [30]. In the present study, we identified a total of 461 proteins in samples collected immediately after parturition. Differences between our observations and values reported by others may be related to sampling day after calving (thus change in milk protein composition) or number of lactations. In this experiment, we sampled cows only on the day of calving and all cows were primiparous. These finding are also evidence of the progress achieved in sample processing technologies and proteomic databases in recent years.

Reports have shown that most proteins in bovine colostrum are involved in major pathways associated with immune and enzymatic activity [29, 31]. In particular, the colostrum proteome is rich in proteins involved in immune and complement cascade activation as well as mineral and vitamin binding activity [29, 30, 32, 33]. In the present study, results agree with prior findings regarding predominant protein classes with crucial roles in main biological processes and metabolic pathways [29, 31]. For example, we found that the complement C3 (Q2UVX4) protein was among the most abundant proteins in the samples. This protein plays a central and versatile role in the activation of the complement cascade system; not only to clear pathogens but also for a variety of homeostatic processes ranging from tissue regeneration and synapse pruning to clearing debris and controlling tumor cell progression [34]. Additionally, the conversion of this protein by the enzyme convertase is the pivotal reaction in both the classical an alternative complement pathways [35]. After its activation, the portion identified as C3b can then bind to cell surface carbohydrates or immune aggregates [36]. Due to its versatility and crucial function in the innate immunity, some researchers have dubbed this protein as the “Swiss army knife” of innate immunity and host defense [37]. In the present study, we found that the complement C3 (Q2UVX4) was concomitantly enhanced with other proteins of the component cascade such as component C4 (A0AAF6ZHP5), and component C6 (F1MM86) in the samples of excellent quality. This indicates that the excellent quality colostrum was indeed superior to other categories by higher IgG concentrations and by increased abundance of those important immune-related proteins, which confirmed our hypothesis.

Additionally, the polymeric immunoglobulin receptor (PIGR; P81265) was among predominant proteins in the colostrum samples. This protein plays a unique role in the mucosal immune system, which acts both as an epithelial transporter and as an integral component of secretory immunoglobulins. In particular, the PIGR is an important precursor of the secretory component [38], which is essential in the transport of IgM and IgA into the colostrum via secretory IgM (sIgM) and secretory IgA (sIgA) [39]. Additionally, the immunoglobulin heavy constant gamma 1 and (IGHG1; X16701_4) was also found to be among most abundant proteins, being an important member of the IgG subtype and a critical functional isoform of immunoglobulins produced by the immune system, with a major role in the innate response and protection [40].

The analysis showed that albumin (A0A140T897) was another abundant protein in samples. This protein is involved in maintaining the oncotic pressure before its release into the colostrum [41]. Reports have also indicated that albumin is closely involved in the transport of molecules and its presence in colostrum is useful to monitor the blood-milk barrier permeability and the transition from colostrum to mature milk [42]. In terms of health function, albumin can act as an anti-oxidant and hence helps in regulating the oxidation–reduction process as part of an anti-inflammatory response [43]. Furthermore, albumin may be part of the mammary gland innate immune system because the components of albumin metabolism increase in response to endotoxin-induced inflammation [43].

For adequate transfer of passive immunity from the dam to the calf, the antibodies of colostrum must be protected from cleavage before being absorbed by the enterocytes [32, 44], which is mainly achieved via the presence of protease inhibitors. Previous studies [9, 45] have reported the presence of a few protease inhibitors in bovine colostrum. This study now identified a wide range of proteins involved in this process; thus, improving the current knowledge. For example, there were several proteins acting as protease inhibitors, which belong to the class of protein-binding activity modulator. Within these protease inhibitors, trypsin and chymotrypsin inhibitors were found to be the most abundant, such as the serpin A3-7 (A2I7N3) and serpin A3-5 (A2I7N1). These are known to act against chymotrypsin [46, 47], and thereby enhance the protection of colostrum proteins at the duodenal level before absorption. In fact, serpin A3 (A2I7N3), is also called α-1-antichymotrypsin, underling its role in protection from digestion. Moreover, it also plays an important role in the anti-inflammatory and antiviral response [48, 49]. Interestingly, we observed that a greater concentration of IgG in colostrum was associated with enrichment of the pathway for regulation of endopeptidase activity. Therefore, findings suggest improved protection of the colostrum proteins against cleavage before being absorbed by the enterocytes by the calves when consuming colostrum of excellent quality.

It is also worth noting that the excellent quality colostrum was rich in IgG and sIgM. Both IgG and IgM are activators of the complement cascade [50]. The IgG-induced activation of the complement cascade plays a major role in the host defense against pathogens. It initiates a robust and efficient proteolytic cascade, which leads to lysis and elimination of the pathogen as well as the generation of the classical inflammatory response through the production of potent proinflammatory molecules [51, 52]. Additionally, the CD5 molecule like (also known apoptosis inhibitor of macrophage; A6QNW7) is a component of the IgM structure [53], which is a crucial component during the calf immune response. This protein was found to be enriched in the colostrum of excellent quality. Researchers have further indicated that the IgM is not only a major initiator of the humoral immune response, but it is also responsible for maintaining immune homeostasis through the induction of tolerance and for clearance of apoptotic cells [53]. Therefore, these findings underscore the positive implications of consumption of good quality colostrum on neonate calves for adequate immune function and resilience against potential pathogenic microorganisms.

This study detected several proteins that possess antioxidant activity as well, namely hemoglobin subunit beta (P02070), glutathione peroxidase (A0AAA9TU07), peroxiredoxin-2 (A0AAA9RUA3), hemoglobin subunit zeta (E1BAP8), and peroxiredoxin-4 (Q9BGI2). These proteins have critical and specific roles in the antioxidative process. For example, glutathione peroxidase (A0AAA9TU07) has been shown to protect cells and enzymes from oxidative damage by catalyzing the reduction of oxidative molecules such as hydrogen peroxide, lipid peroxides and organic hydroperoxide [30, 54]. Likewise, peroxiredoxin-4 (Q9BGI2) catalyzes the reduction of hydrogen peroxide to water, a process that results in the protection of cells against oxidative stress [55]. Protection of the calf against oxidative stress is of vital importance to prevent damage of tissue, especially at young age when the neonate is more vulnerable to health complications [56]. Therefore, these key proteins may contribute to set the newborn calf to a good start in life. Our findings further showed that a greater content of IgG in colostrum may result in decreased oxidative stress as shown by the lower abundance of xanthine dehydrogenase/oxidase (P80457) in samples of excellent quality. This enzyme has been shown to influence the oxidative stress status in animals because of its contribution to the generation of superoxide ion, hydrogen peroxide, and nitric oxide [57].

One of the factors that has been suggested to impair the successful transfer of passive immunity in calves is the presence of certain pathogenic bacteria that bind IgG in the gut [58]; thus, decreasing their availability for absorption by the enterocytes. Another suggested mechanism for impaired absorption of IgG is through interference of certain bacteria at the absorption sites [59]. This study revealed the presence of several proteins with antimicrobial properties in bovine colostrum. For example, xanthine dehydrogenase/oxidase (P80457) and cathelicidin-4 (A0AAF6ZFN1), belonging to the protein class for antimicrobial response protein, have been reported to display a defense response against a broad spectrum of bacteria [30, 60–62]. In addition, lactoperoxidase (P80025), has been suggested to have antioxidant activity that contributes to protection against microorganisms. Specifically, this protein catalyzes the oxidation of thiocyanates in the presence of hydrogen peroxide; which leads to the generation of compounds with antimicrobial action [63, 64]. Therefore, research on early life dietary managements to improve the presence of proteins possessing antimicrobial properties in colostrum may be reflected in better transfer of passive immunity from the dam to the newborn. In addition, lactotransferrin (P24627) has been reported to bind iron, resulting in the free iron being sequestered and rendered unavailable for bacterial utilization and growth; thus, lactotransferrin (P24627) exerts a bacteriostatic effect against potential pathogens, which may contribute positively to development of the newborn [37, 65, 66]. Lactotransferrin (P24627) can bind to lipopolysaccharide of bacterial cells as well, and may damage bacteria via formation of peroxides catalyzed by lactotransferrin-bound iron, affecting membrane permeability and resulting in bacterial cell lysis [37, 65, 66].

Overall, the differences in abundance of colostrum proteins associated with IgG concentration were reflected in enrichment primarily of metabolic pathways and biological processes associated with immune response and complement activation, which contribute to the protection of the neonate against possible pathogenic microorganisms. Whereby, the protein interaction analysis revealed a close interplay among proteins of the complement component cascade and cofactors. For example, complement component C3 (Q2UVX4) acts as a point of convergence of activation pathways and helps to coordinate downstream immune responses against pathogens [34]. Thus, results indicate that increased IgG content is associated with improved colostrum properties contributing to better health of neonates, and support other reports suggesting that colostrum quality influences the proteomic profile [28]. However, our findings also reveal that the increased presence of IgG in colostrum was associated with a reduction of a few proteins such as beta-casein (A0A452DHW7) and alpha casein-S1 (P02662), two of the most abundant proteins in this study as well as from in reports in colostrum [29, 67, 68] or milk [30, 32, 69]. Alpha casein-S1 (P02662) has been shown to influence abundance of other proteins in the mammary gland [69] as well as having crucial roles in neonatal development in other animal species [70]. Nonetheless, the implications of the lower content of casein with greater content of IgG in the bovine colostrum deserves further investigation to clarify whether those changes have implications on the supply of nutrients and other compounds to the calf.

Previous research has suggested that pre-calving cows’ blood proteins translocate into the mammary gland for colostrogenesis [71], which may influence colostrum composition. In this study, correlation analysis indicated that greater pre-calving blood globulin content of cows was associated with greater IGKV2-18*01 (IMGT000047_11), haptoglobin (Q2TBU0) and lactotransferrin (P24627) in the colostrum. As outlined before, these proteins play roles in immune protection and mineral binding [72–74]. In addition, calf passive immunity correlated with serpin family G member 1 (A0AAA9TZ37) and with cathelicidin-7 (P56425) in colostrum. Serpin family G member 1 (A0AAA9TZ37) is a protease inhibitor [75]; while cathelicidin-7 (P56425) acts as an antimicrobial peptide [76]. On the other hand, selenoprotein F (A8YXY3), involved in cell signaling and immune modulation, negatively correlated with calf passive immunity [77]. These correlations suggest the potential influence of the nutrition and blood composition of the cow on the colostrum proteome and eventually on the health of the calf, a topic that deserves further in-depth research. In addition, future studies should evaluate the quality of colostrum consumed by calves in the days following parturition to further investigate the role of colostrum proteins on passive immunity transfer to the calves.

## Conclusions

Our research enriches the understanding of the bovine colostrum proteome, highlighting the role of key colostrum proteins in biological processes such as cellular process, biological regulation, response to stimulus, metabolic process, and immune system process. Most abundant proteins included complement C3 (Q2UVX4), alpha-S1-casein (P02662), Ig-like domain-containing protein (A0A3Q1M032), albumin (A0A140T897), PIGR (P81265), and lactotransferrin (P24627). Colostrum of excellent quality contained greater abundance of several key proteins such as serpin A3-7 (A2I7N3), complement factor I (A0A3Q1MIF4), lipocalin/cytosolic fatty-acid binding domain-containing protein (A0A3Q1MRQ2), and complement C3 (Q2UVX4). This resulted in enrichment mainly of the biological processes for complement activation alternative pathway, complement activation, complement activation classical pathway, and humoral immune response. Additionally, greater quality of colostrum leads to an increase in antimicrobial and antiprotease activities, indicating enhanced protective properties of colostrum prior to its absorption in the calf’s gut. Results revealed that higher levels of colostrum IgG led to reduced beta and alpha casein, as well as proteins linked to oxidative stress. Therefore, the content of colostrum IgG was strongly associated with the abundance of other important immune-related proteins and may be a suitable indicator for categorizing the proteome profile. Our observations underscore the importance of consumption of colostrum of excellent quality for a better response against pathogenic microorganisms. Future research should deeply explore the association of these findings with the nutrition status and blood composition of the cow as well as with the passive immunity transfer in the calf.

## Supplementary Information

Additional file 1. Hierarchical cluster dendrogam showing good correspondence between each pair of technical replicates for the bovine colostrum samples. This dendrogram was constructed using the abundance values of proteins within the entire proteome as an input.

## Data Availability

Additional data and material of raw proteomic output for this paper were deposited on Mendeley data [22].

## Abbreviations

ACN: Acetonitrile
DTT: Dithiothreitol
GO: Gene Ontology
HPLC: High performance liquid chromatography
IAA: Alkylated in iodoacetamide
IgG: Immunoglobulin G
LC–MS/MS: Liquid chromatography coupled to mass spectrometry
NCF: Non-fiber carbohydrates
PBS: Phosphate buffered saline
SDS-PAGE: Sodium dodecyl sulfate–polyacrylamide gel electrophoresis
SRID: Single radial immunodiffusion
TFA: Trifluoroacetic acid
TRIS: Tris (hydroxymethyl) aminomethane
