# Supplementary material for: Characterization of the colostrum proteome of primiparous Holstein cows and its association with colostrum immunoglobulin G concentrations
Source: J Anim Sci Biotechnol. 2025 Jan 21;16:10. doi: 10.1186/s40104-024-01144-y (PMC11748342; doi:10.1186/s40104-024-01144-y)
Supplement: Supplementary file 1 — Additional file 1. Hierarchical cluster dendrogam showing good correspondence between each pair of technical replicates for the bovine colostrum samples. This dendrogram was constructed using the abundance values of proteins within the entire proteome as an input. [file 40104_2024_1144_MOESM1_ESM.docx]

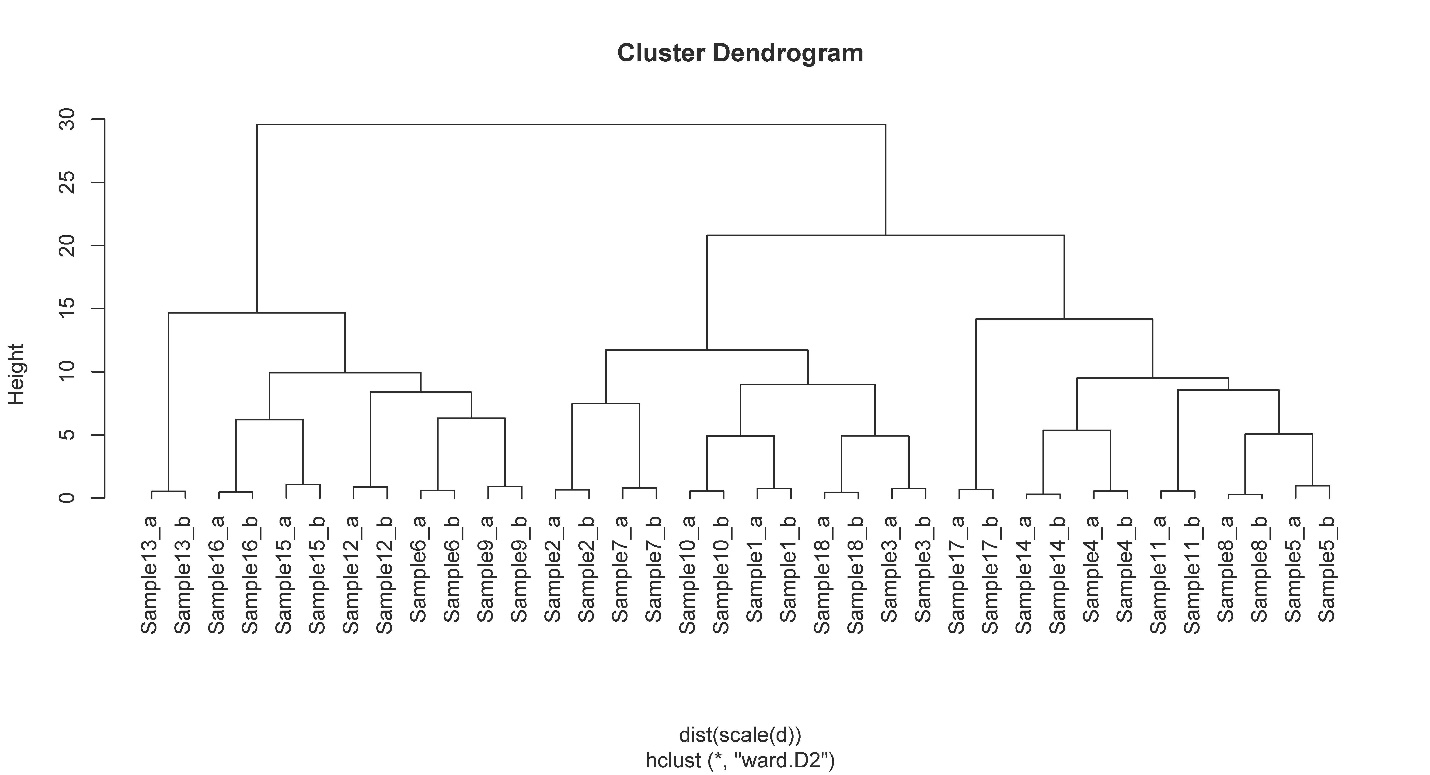


Hierarchical cluster dendrogam showing good correspondence between each pair of technical replicates for the bovine colostrum samples. This dendrogram was constructed using the abundance values of proteins within the entire proteome as an input.
